# Two Mechanisms of Sensorimotor Set Adaptation to Inclined Stance

**DOI:** 10.3389/fnhum.2017.00480

**Published:** 2017-10-23

**Authors:** Kyoung-Hyun Lee, Asheeba Baksh, Alyssa Bryant, Mollie McGowan, Ryan McMillan, Raymond K. Chong

**Affiliations:** ^1^Center for Sport Science in Gwangju, Gwangju Sports Council, Gwangju, South Korea; ^2^Department of Physical Therapy, Augusta University, Augusta, GA, United States

**Keywords:** balance control, postural control, sensorimotor set, somatosensory, vestibular, vision

## Abstract

Orientation of posture relative to the environment depends on the contributions from the somatosensory, vestibular, and visual systems mixed in varying proportions to produce a sensorimotor set. Here, we probed the sensorimotor set composition using a postural adaptation task in which healthy adults stood on an inclined surface for 3 min. Upon returning to a horizontal surface, participants displayed a range of postural orientations – from an aftereffect that consisted of a large forward postural lean to an upright stance with little or no aftereffect. It has been hypothesized that the post-incline postural change depends on each individual’s sensorimotor set: whether the set was dominated by the somatosensory or vestibular system: Somatosensory dominance would cause the lean aftereffect whereas vestibular dominance should steer stance posture toward upright orientation. We investigated the individuals who displayed somatosensory dominance by manipulating their attention to spatial orientation. We introduced a distraction condition in which subjects concurrently performed a difficult arithmetic subtraction task. This manipulation altered the time course of their post-incline aftereffect. When not distracted, participants returned to upright stance within the 3-min period. However, they continued leaning forward when distracted. These results suggest that the mechanism of sensorimotor set adaptation to inclined stance comprises at least two components. The first component reflects the dominant contribution from the somatosensory system. Since the postural lean was observed among these subjects even when they were not distracted, it suggests that the aftereffect is difficult to overcome. The second component includes a covert attentional component which manifests as the dissipation of the aftereffect and the return of posture to upright orientation.

## Introduction

An ongoing challenge for researchers in the field of postural control is understanding how people combine their senses, namely the vestibular, somatosensory, and visual systems in different proportions to achieve the same outcome behavior. The differences may come from any number of sources, including age, lifestyle experiences, postural orientation, sensorimotor set (based on immediate prior experience), intrinsic perceptual–motor variability, perception of task requirement, etc. In laboratory studies, the directives of the researcher may also influence the test participants’ postural responses (Peterka and Black, 1990; Horak and Macpherson, 1996).

Here, we investigated an experimental protocol in which participants exhibit a response which dissipates over time. Subject’s sensorimotor set is first primed by standing on an inclined surface for several minutes with eyes closed. Upon returning to a level surface, a range of postural responses can be observed in the post-inclined phase, from an ordinary upright posture to a body lean, mostly at the ankles. The amount of lean is referred to as the aftereffect. It reflects an alteration in the sensorimotor set based on prior experience with the inclined stance. The aftereffect dissipates over time, and participants eventually return to upright posture (Kluzik et al., 2005).

It has been hypothesized that those individuals who show the aftereffect in the post-inclined phase align their postural orientation based on a dominant somatosensory system while those who do not are aligning themselves with a dominant vestibular system (Kluzik et al., 2005). Alignment with the somatosensory system would mean that participants lean forward to reproduce the ankle angle encountered during the inclined stance. On the other hand, aligning with the vestibular system would be expressed as a constant upright postural orientation to coincide with the gravitational vector.

The lean aftereffect can be consistently observed within participants during repeated testing and in different directions of sloped stance (Kluzik et al., 2005). It appears to originate at least in part within the central nervous system. When participants’ lower extremities were prevented from leaning, their unconstrained trunk continued to display the aftereffect (Kluzik et al., 2007). Varying the angle of incline influenced the aftereffect in the trunk and head but not in the lower extremities. Affording participants the benefit of visual inputs also did not hasten the recovery from the aftereffect. Upright posture was temporarily restored when participants’ blindfolds were removed, but the aftereffect resumed its influence when vision was again taken out. In some cases, the magnitude of postural lean increased more than in participants who remained blindfolded throughout testing (Earhart et al., 2010). These studies suggest that the aftereffect, which reflects the newly configured sensorimotor set is difficult to overcome.

Stance postural control may also be influenced by the subject’s awareness of the task in the context of what they should be doing (Stoffregen and Riccio, 1988). Based on past experience, an abstraction of the task (the goal, the consequence, etc.) is incorporated into the coordination of the current action (Posner and Keele, 1968; Horak, 1996; Chong et al., 1999, 2000). For example, when standing on a train that is about to move, one may prepare for an impending loss of balance by holding tightly onto the grab bar and widening the stance. This perceptual–motor system interaction may be self-initiated: understanding the consequence of not supporting oneself, or influenced by instructions: warning by the train operator.

Here, we asked how priming the sensorimotor set with the inclined stance impacts the time course for normalization in the presence of a cognitive challenge. We asked whether awareness of spatial orientation may influence the duration of the normalization period. We hypothesize that the perceptual–motor system, specifically awareness of one’s postural orientation, plays a role in the adaptation of the sensorimotor set. For example, in prism finger-pointing studies, it has been found that some individuals sourced a predominantly cognitive strategy to overcome the prism aftereffect by correcting the trajectory of the pointing limb (Redding and Wallace, 1996). The correction occurs because the prism lens distorts the target’s location and the subject corrects their trajectory after they realized their mistake. The adjustment is thought to entail a conscious effort to defeat the aftereffect simply because the subject is aware that their limb was moving incorrectly.

In the inclined stance protocol, there is also evidence that some participants may have been aware of their lean aftereffect and countered it by attempting to stand upright (Kluzik et al., 2005; Regia-Corte and Wagman, 2008; Chong et al., 2016). Whether this was done consciously is part of what will be investigated in the current study. Our overall hypothesis is that a concurrent cognitive challenge will prolong the eventual return to upright stance.

## Materials and Methods

### Participants

A convenience sample of 40 healthy adults (20 men and 20 women) between 21 and 34 years old (mean 25 ± 2 years) reporting no significant neurological or musculoskeletal impairments were recruited to participate in the study which was approved by the Augusta University Institutional Review Board. The experiment was carried out with the understanding and written consent of each subject. None of them had prior experience with the test protocol.

### Common Procedures

Participants wore comfortable flat shoes and a safety body harness strapped to a fixed overhead frame. The harness had sufficient slack to allow free body movements. Their feet were outlined to ensure consistency between conditions. Participants stood with arms by the sides in quiet stance on a force platform sampled at 100 Hz (NeuroCom International, Clackamas, Oregon). They wore a blindfold to aid eye closure throughout testing. During testing, the antero-posterior center of pressure (A/P CoP) representing each subject’s body sway was recorded. The force data were processed following the methods from previous studies. Frequencies above 0.1 Hz were filtered out. A/P CoP pre-inclined sway (quiet stance, eyes open) was averaged over 30-s and subtracted from the post-inclined A/P CoP sway data to reveal the lean aftereffect component (i.e., the gradual return to upright stance) thereby distinguishing it from higher-frequency movements comprising the back-and-forth corrections of postural sway (Kluzik et al., 2005; Kouzaki and Masani, 2012).

At the end of the experiment, participants were surveyed regarding whether they were aware of their postural orientation during the post-inclined phase and what they did, if anything. Participants were asked to choose a response that corresponded with their perception of postural orientation after each condition, as follows: (1) Yes definitely, or quite sure of leaning, (2) Not sure, felt like swaying but not sure if leaning, (3) No, felt like swaying, but not aware of leaning, and (4) Other response (asked for a subjective description).

### Experimental Conditions

Participants were tested in two cognitive conditions. In the No-Distraction condition, participants were tested in the protocol described above. In the Distraction condition, participants were instructed to subtract by seven non-stop from a given number during the post-inclined phase. The subtraction task was used because other methods of distraction appear to produce less than maximal distraction, such as word generation (Chong et al., 2010). The same number was used for every subject. Maximal distraction was further induced by instructing participants to count as quickly and as accurately as possible and to not correct themselves if they gave an incorrect answer (Pashler, 1994; Lacour et al., 2008). Scoring a correct or incorrect response was based on the previous answer even if it was incorrect (Chong et al., 2010). Participants rehearsed the counting three to five times (different numbers) to ensure they understood the instructions. Responses were recorded via a handheld digital audio recorder.

**FIGURE 1:**
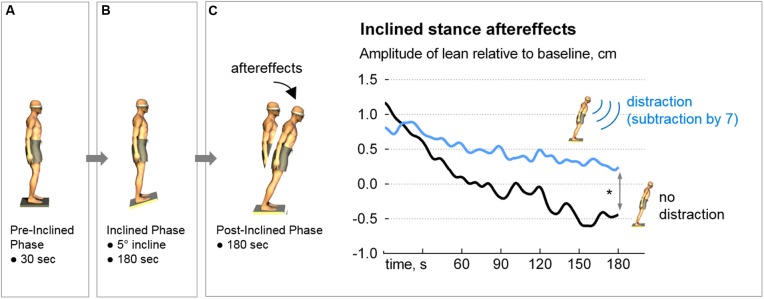
The inclined-stance protocol and results. After baseline sway was performed **(A)**, each participant’s sensorimotor set was primed by standing on a 5-degree toes-up incline for three minutes **(B)**. Postural aftereffect (forward body lean) was then examined under two conditions: with and without distraction (serial 7 subtractions). In the Distraction condition, where the participants subtracted non-stop during the post-inclined phase, recovery from the aftereffect was prolonged (blue trace, **C**), ^∗^*p* = 0.012. Note that the *y*-axis represents the change in anteroposterior sway compared to Pre-Inclined. Therefore, the zero value does not represent a perfectly vertical stance (i.e., parallel to the gravitational vector). Likewise, the negative values do not imply that the participants leaned backward.

Each condition consisted of three phases as follows: (1) Pre-inclined phase: eyes closed, quiet stance for 30 s; (2) Inclined phase: eyes closed, quiet stance on 5° toes-up stiff board for 3 min; and (3) Post-inclined phase: eyes closed, quiet stance on level surface for 3 min (**Figure 1**).

Each experimental condition lasting approximately 10 min was tested once, with a 15-min mandatory seated break between conditions. The order of testing was randomized among the participants; half started with the No-Distraction condition.

### Analyses

Participants’ postural sway outcomes were analyzed using a 2 (Period) × 2 (Distraction) repeated measures ANOVA comparing the initial average A/P sway (the first 5 s) and end (last 5 s) period of the two conditions. Responses to the inclined stance were classified as positive, indicating the presence of an aftereffect if the A/P sway at the beginning and end of the post-inclined phase was different. The pattern of the aftereffect was determined to be either linear and exponential with the least-square-fit regression test (Kluzik et al., 2007; Chong et al., 2014). The speed (number of responses per minute) and accuracy (percentage of total responses) of the subtraction task were also analyzed with a two-way repeated measures ANOVA. Alpha was set to 0.05. A significant interaction effect was followed up with a Bonferroni *post hoc* simple-effects test.

## Results

### Influence of Concurrent Distraction on the Post-Inclined Aftereffect

Eighty-five percent of the participants (*n* = 34, hereafter referred to as responders) showed the lean aftereffect, whereas 15% (*n* = 6, non-responders) did not. Fifteen participants (44%) had a linear-type adaptation. Nineteen participants (56%) displayed the first-order decay-type adaptation. Their average fitting function results are summarized in **Table 1**. The coefficient of determination, *R*^2^ representing the proportion of the variance in the aftereffect which is explained by the predictor variable (time) was 0.45 ± 0.17 in the No-Distraction condition and 0.40 ± 0.21 in the Distraction, similar to a previous report (Chong et al., 2014).

**Table 1:** Summary of the curve fitting exponential function among responder participants who displayed the first-order decay pattern of adaptation.

	No-Distraction condition, mean (*SD*)	Distraction condition, mean (*SD*)
*Y*_0_ (cm)	1.2 (1.7)	0.8 (1.5)
Plateau (cm)	-0.5 (1.2)	0.9 (2.2)
*K* (s^-1^)	0.1 (0.3)	0.2 (0.4)
Half-life (s)	32.5 (63.3)	64.4 (102.5)
Tau (s)	46.9 (91.4)	92.9 (147.9)
Span (cm)	1.7 (1.8)	-0.1 (3.0)


*Note that many participants did not normalize their posture at the end of the 180-s post-inclined phase in the Distraction condition. The results should therefore not be interpreted verbatim. Y_0_, initial value of the one-phase decay; Plateau, steady state of the decay (not constraint to zero); K, rate constant; Half-life, computed as ln(2)/K; Tau, time constant; Span, difference between Y_0_ and Plateau*.

The interaction between the Period and Distraction conditions was significant, *F*(1,33) = 19.01, *p* = 0.0001. The anterior displacement of the A/P CoP at the beginning of the post-inclined phase was similar between the No-Distraction and Distraction conditions (1.1 ± 1.4 vs. 0.8 ± 1.2 cm, *p* = 0.21). At the end of the 180-s post-inclined phase, participants had resumed upright stance when they were not distracted (No-Distraction condition) whereas they remained leaning when distracted (-0.5 ± 1.2 vs 0.2 ± 1.3 cm, *p* = 0.02). This resulted in a smaller span (range of postural lean) in the Distraction condition (1.7 ± 1.2 vs. 1.04 ± 0.8 cm, *p* = 0.012, **Figure 1C**).

### Perception of Postural Lean

Among the responders, seven participants reported awareness of the forward lean in the Distraction condition only; five were cognizant in the No-Distraction condition only and one subject was aware of it in both conditions.

Of the six non-responders, only one subject thought that they were leaning forward in both conditions. One subject was aware of the lean in the Distraction but not the No-Distraction condition. Both of them recalled that they willfully corrected their lean. A summary of the participants’ responses to the post-inclined survey is presented in **Table 2**.

**Table 2:** Perception of forward lean.

	Count No-Distraction, Distraction	Example comments by participants
(1) Quite sure leaning forward		R =
	*R* = 6, 2	B: “I felt like I swayed more”
		B: “I know I put a lot of weight through my toes”
	NR = 1, 2	NR =
		D: “I moved a little, but my balance was pretty good … I did better on the first one, but got bored on the second and had more sway”
(2) Not sure if leaning		R =
		B: “I still felt like I did some”
		D: “I felt okay … a little bit”
	*R* = 8, 12	D: I think I did fine … I feel like I swayed some”
	NR = 3, 1	D: “I felt like I was swaying a little bit”
		D: “I was aware of leaning and corrected myself”
		NR =
		B: “I felt a little swaying”
(3) Not aware of leaning		D =
		“I tried not to sway … I don’t think I leaned in one, I swayed in both”
		“I knew I was swaying”
	*R* = 15, 15	B: “I swayed a little bit”
	NR = 2, 2	D: “I felt like I did better, but I was still swaying a little bit”
		D: “I was less this time because I was focused on counting”
		NR = B: “I like changed where my weight was distributed sometimes … I did not feel like I was leaning”
(4) Other		R =
		“I wasn’t aware of swaying or leaning on either trial”
		“didn’t feel swaying or leaning”
		B: “I was pushing more into my feet so I wouldn’t lean … I felt like I was pushing down with the front of my feet to prevent leaning”
	*R* = 5, 5	B: I didn’t feel I was leaning, maybe leaning to the right a little because my left leg is longer”
	NR = 0, 1	D: “I was just thinking about counting … I didn’t feel anything … I was too busy concentrating on counting”
		D: “I felt like it’s easier to balance when you’re counting … I didn’t feel like I was”
		D: “I probably swayed more the second time since I was trying to concentrate”
		NR =
		D: “I don’t think I was moving at all”


*Participants were asked whether they were aware of how their body was moving during the Post-Inclined phase. The choice of answers was as follows: (1) Yes definitely, or quite sure of leaning, (2) Not sure, felt like swaying but not sure if leaning, (3) No, felt like swaying, but not aware of leaning, and (4) Other response (asked for a subjective description). R = responders; NR = non-responders; B: no-distraction condition; D: distraction condition (concurrent serial subtraction by 7); Distraction = concurrent serial backward subtraction by 7; and No-distraction = quiet stance, no subtraction activity*.

### Cognitive Performance

Responders and non-responders performed similarly in the subtraction task (*p* > 0.05). The average accuracy and speed of subtraction was 85 ± 12 and 15 ± 7% responses per minute, respectively.

## Discussion

### General Discussion

The results of the study showed that while the responder participants leaned by the same amount initially in the two cognitive conditions following the inclined stance, cognitive distraction resulted in an incomplete dissipation of the lean aftereffect. The frequency of responders and the nature of their aftereffect profile (first-order and linear sway trajectory) are comparable to previous findings (Kluzik et al., 2005; Chong et al., 2014). These findings are consistent with previous work, in that the adaptation of the sensorimotor set is an obligatory neural process that is difficult to hasten or overcome without vision or cognitive provisions (Teasdale et al., 1991; Kluzik et al., 2007; Earhart et al., 2010). Although postural control requires some executive control, it is not fully amenable to volition (Chong et al., 1999, 2000).

Although the responder participants in the Distraction condition had not recovered from the aftereffect after 180 s in the post-inclined phase, their exponential decay pattern is in contrast to their No-Distraction pattern. Their time constant and half-life are longer than the No-Distraction in the current and previous studies (Kluzik et al., 2007; Chong et al., 2014) while their span or range of sway between the first and last 5 s is smaller. The differential between the two cognitive conditions was 1 cm. Although this appears to be a small difference, a previous study showed that standing on higher inclines produce correspondingly larger aftereffect (Kluzik et al., 2007). Participants underwent four inclined-amplitude conditions: 2.5°, 5°, 7.5°, and 10°. The maximum lean was found to level off at the 7.5° and 10° inclinations, suggesting that participants can increase their aftereffect but will not go past a certain point to prevent a loss of balance. Therefore, if we had used an inclined greater than 5°, we should observe a larger aftereffect.

Next, we consider three non-mutually exclusive possible explanations to account for the observation of the delayed dissipation of the aftereffect among the responders in the Distraction condition. The first is regarding the choice of the distractor task. We previously showed that performing a difficult subtraction task interfered with stance balance control more so than a verbal fluency (word generation) task (Chong et al., 2010). Many people manage a difficult mental task by imagining it. In the case of the serial seven subtractions, people visualize the calculations in their head. Visualization may explain why people sometimes look to the side or close their eyes to better recall an event. Visual mental calculations and balance control are thought to use the same parts of the brain (Chong et al., 2010). According to the modular theory of motor control (Chong et al., 2010), if two such activities are performed in parallel, a performance cost may occur in which the efficiency of either one or both tasks is compromised. In the current study, the serial subtraction task may have interacted with postural orientation via common activations of the parietal region (Chong et al., 2010). The extended duration of the aftereffect would be the manifestation of it. The source of the interference is considered a neural and not a physical phenomenon because the subtraction task is a mental activity whereas the inclined stance is a postural (physical) deed. Thus, the observation of prolonged aftereffect cannot be explained by the constraint of attempting to execute two physical (bodily) tasks concomitantly (Chong et al., 2010).

Secondly, since some amount of attention needs to be devoted to maintaining one’s balance control (Woollacott and Shumway-Cook, 2002), it may be the case that the participants perceived being able to manage their postural lean, not feel threatened and therefore not return to upright stance. For example, Doumas et al. (2008) asked their participants to keep their balance while working out an *n*-back task concurrently. In the elderly group, when standing on an unmoving surface, body sway increased compared to simply standing without performing the *n*-back task. On the other hand, when they stood on a pliable surface, body sway was the same between the single and dual-task conditions. The authors suggested that people sacrificed their balance control and “allow” their body sway to increase if it would not cause them to lose their balance (as in standing on a firm surface) in order to devote more attention to the cognitive task. Whether the participants in our study allowed themselves to remain in the forward lean or not during the distraction condition needs further experimentation.

Thirdly, the results of an earlier study suggest that the somatosensory component of the inclined stance (the other component being the vestibular system as described in the section “Introduction”) may also partly explain the prolonged aftereffect observed in our cognitive distraction condition. In that study, participants were asked to lightly touch a rigid board with their fingertips during the inclined-stance phase (Chong et al., 2014). The purpose was to see if enhancing somatosensory inputs would increase the aftereffect. The results showed that adding light touch to the inclined stance protocol increased the number of responders (i.e., more leaners) compared to the no-touch condition. In addition, some of the responders remained leaning far forward throughout the 3-min post-inclined phase, an unusual response that has never been reported.

The distraction task in the current study appeared to have influenced sensorimotor set significantly. The somatosensory system may have become more dominant like the earlier study described above (Chong et al., 2014), expressing itself with minimal cognitive intervention, particularly in the later period of the post-inclined phase (Borel et al., 2008). Increased influence of the somatosensory system was also suggested in individuals with a vestibular-related disorder. Following prolonged stance on an inclined surface, all the participants in the study displayed the lean aftereffect (Chong et al., 2016). We are studying individuals with somatosensory loss to further understand the source(s) of the aftereffect.

Among the small number of non-responders, we cannot rule out the possibility that participants may have willfully corrected their postural lean but not retained their actions. Studies have shown that people can carry out a voluntary activity repeatedly (with or without distraction) and yet not recognized that they had implicitly acquired a new movement structure (Keele et al., 2003).

### Perception of Body Sway

Since many of our participants reported little or no overt awareness of their postural lean, the response to the inclined stance which produced the lean aftereffect appeared to encompass a different neural domain, one that involves a more automatic sensory integrative process that is driven by the somatosensory system. Thus, we assumed that participants were either adequately allocating their attention or were able to switch back and forth between the subtraction and the automatic postural adaptation of the aftereffect (Laberge, 1981). If so, then one might argue that the sensorimotor set in the Distraction condition should be similar to the No-Distraction condition. Indeed participants in both conditions started out leaning forward by the same amount. Toward the end of the trial however, participants in the Distraction condition continued to display the aftereffect, suggesting that the time course for sensorimotor set to normalize was different between the two conditions.

We noted that some non-responder participants (i.e., non-leaners) reported the sensation of leaning while some responders (leaners) claimed no such impression. It may be that for these responders, the slow postural drift associated with the aftereffect was obscured from awareness by the higher frequencies postural sway which occurs during quiet stance, especially during the transition from one postural sensory condition to another (Teasdale et al., 1993; Teasdale and Simoneau, 2001). The non-responders, on the other hand, may have mistaken the higher frequencies sway to be a feeling of leaning. The difficulty in differentiating the wobbling from the minute body sway adjustments versus the embedded slower postural drift of the aftereffect may explain these seeming discrepancies in perception of body motions (Garkavenko et al., 2012; Kouzaki and Masani, 2012).

The subtraction condition utilized in the current study was designed to both maximally distract the participants and create an impediment to the sensorimotor integrative mechanisms. A previous study had examined balance control performance while concurrently performing one of two mental activities. One was subtracting backward by seven and the other was generating words of the same first letter. The balance control task was performed under three levels of visuospatial difficulty. The subtraction task contributed to a decrease in balance control to a greater degree than the word generation task. There was also a decrease in the speed and accuracy of responses in the distraction task. The authors suggested that sensory organization for balance control appears to require similar visuospatial resources for the subtraction task, but not the word generation task (Chong et al., 2010). Another study attempted to do something similar, but it is not clear whether the objective of maximal distraction was achieved (Kluzik et al., 2007). Their participants were asked to listen to an undefined story while undergoing the inclined stance protocol. Although the purpose of listening to the story was to distract the participants, they were also instructed not to resist their body lean during the post-inclined phase if they felt it. It is possible that this instruction may have defeated the purpose of listening to the story. It may have preordained the participants to detect changes in their postural orientation. We cannot be sure that this happened to at least some of their participants because they were not debriefed about it. Moreover, their participants were not tested on their ability to recall the story they heard. It is also not clear whether participants were asked to remember the details of the story, which would have increased the confidence that they were substantially distracted.

### Application

Although the rationale behind this study was to determine the effect of mental distraction on the sensorimotor set of healthy participants, the results of this study may be clinically applicable. Unlike many studies, the inclined stance protocol allows us to study a form of adaptation which takes place over a relatively long period. The results suggest that postural adaptation may comprise a voluntary component which may act without awareness, and when paired with a mental task, the distraction produces an extended postural adaptation. These findings can be important in regards to both physical therapy assessments and treatment interventions. In regards to assessments, it is important to ensure that patients are focused on the task at hand because an effective distraction task could possibly contribute to inaccurate results of the assessment. For example, when assessing an individual’s shoulder flexion strength, it is important to ensure that the individual is focused on maintaining the proper manual muscle testing position while the assessment is being performed in order to prevent inaccurate results.

In a real-world setting, it is common for attention to be diverted during tasks of daily living. Whether individuals are talking on the phone, taking care of children, or thinking about the next meal, people are not always focused on their balance or posture. This is why it is important to understand how the concept of distraction can be incorporated into physical therapy treatment interventions, especially for people experiencing difficulties with their balance. Individuals who have a somatosensory dominance without major influences from the visual or vestibular systems may experience difficulties with balance when they are distracted. Therefore, the concept of distraction tasks can be incorporated into treatment interventions by focusing on awareness of leaning and postural adaptation during distraction tasks. Interventions aimed at increasing awareness of leaning during distraction tasks could possibly contribute to decreasing the risk of falling.

Along with using distraction tasks as a component of treatment interventions, the results of this study may also be useful in determining the reasons as to why individuals are experiencing balance-related difficulties. Along with implementing distraction tasks as a treatment intervention, it may also be useful to remove distraction tasks in order for individuals to become more aware of their leaning and increase their postural adaptation to prevent falls. After increasing awareness of leaning, it may be beneficial to then progress to more functional activities that require dual-tasking. This concept of progressing treatment interventions from no distraction task to the incorporation of a concurrent distraction task can be associated with Gentiles Taxonomy.

## Conclusion

The results suggest that mechanism of sensorimotor set adaptation to inclined stance comprises at least two components. The initial immediate body lean observed among responder participants in the post-inclined phase appears to be an obligatory (true) aftereffect of the inclined stance that is difficult to defeat. This was the case whether or not participants were maximally distracted by the concurrent and challenging cognitive task. The second component involving the normalization of the sensorimotor set via gradual return to upright stance includes a covert attentional component.

## Author Contributions

All authors listed have made a substantial, direct and intellectual contribution to the work, and approved it for publication.

## Conflict of Interest Statement

The authors declare that the research was conducted in the absence of any commercial or financial relationships that could be construed as a potential conflict of interest.
